# Recruitment and selection of community health workers in Iran; a thematic analysis

**DOI:** 10.1186/s12889-023-15797-3

**Published:** 2023-05-09

**Authors:** Sareh Shakerian, Gozal Shafeei Gharanjik

**Affiliations:** Departments of Community Based Education of Health Sciences, Virtual School of Medical Education and Management, Shahid Beheshti University of Medical Sciences, Tehran, Iran

**Keywords:** Recruitment, Selection, Admission, Employment, Community Health Workers, Thematic analysis, Qualitative study

## Abstract

**Background:**

In Iran, community health workers (CHWs) are selected and employed according to the instructions of the Ministry of Health (MOH). The present study aimed to investigate the views of different stakeholders regarding the selection criteria, as well as the competency of CHWs.

**Methods:**

This study was conducted using a qualitative thematic analysis in Golestan Province, Iran. Data were collected using semi-structured interviews with managers, supervisors, CHWs, and common people in 2021. The interviews were recorded and then transcribed. To extract key themes, the six-step Brown model was used, which involved becoming acquainted with the data, meaningful organization of transcripts, extracting primary open codes, searching for themes in an iterative approach, theme extraction, defining themes, and preparing a report. The relationships between codes and sub-themes and themes were represented using ATLAS.ti version 8.

**Results:**

Data saturation was achieved after interviewing 22 people. The extracted data included 340 open codes, two main sub-themes of “CHW effectiveness” and “CHW sustainability”, and three main themes of “criteria for employing competent people”, “barriers to employing competent people”, and “identifying the barriers to employing competent people”, according to the MOH instructions.

**Conclusion:**

In the present study, local hiring was one of the major challenges in the competency-based selection of CHWs. One of the most repeated codes was expanding the local hiring concept and its requirements. Since different regions of Iran have different climatic, economic, cultural, and social conditions, the selection and hiring criteria for CHWs should be tailored to the needs of the community.

## Background

Non-discriminatory and fair access to quality healthcare services for all people around the world is one of the key concepts of the World Health Organization (WHO). To achieve this goal, one of the important health strategies in underprivileged and remote areas is to provide community-based services by community health workers (CHWs) to increase access to quality healthcare services, reduce inequalities, attain justice, and nurture health-promoting lifestyles [1–3]. In recent years, during the COVID-19 pandemic and the global movement toward health justice, the impact of this disease on communities with a low socioeconomic status highlights the substantial role of CHWs in the prevention and control of this disease [4].

The CHWs are professionals or non-professional individuals who work at the forefront of the healthcare system and are generally selected from the community, where they serve their duties; therefore, they have a deep understanding of the language and culture of that particul2qar community. They can overcome many obstacles that hinder service delivery to communities, such as cultural, linguistic, and moral gaps, thereby bridging the formal sectors of the community. These individuals have multiple tasks and roles and act as mediators of health promotion where they provide healthcare services [2, 5–7].

Substantial evidence suggests the cost-effectiveness of providing community-based services by CHWs [8–9]. A study by Kangovi et al. showed that for every dollar invested in community-based interventions by the CHWs, the average return on investment is $2.47 [10].

However, some evidence indicates the low effectiveness of CHWs in the workplace they serve, so they are unable to fulfil their tasks, causing them to withdraw [5, 11]. Therefore, it is necessary for what way to select the CHWs among volunteers to effectively provide community-based services in the workplace, requiring social participation and approval of civil societies.

One of the basic principles of employee selection is to respond to the needs of the community located in that particular region. Residence in the place of service is usually one of the main criteria for employee selection, as emphasized by the WHO in 1989. However, evidence shows that this method of selection may not be ideal or as effective as expected on some occasions [1, 5, 12]. Criteria and policies for selecting and hiring CHWs vary in different communities and countries. In some countries, these selection criteria are determined by the Ministry of Health (MOH) for each local district. In some other countries, national standards and tests are used, regardless of the place of service delivery. In countries, such as India and Ethiopia, these criteria vary from one region to another, depending on the cultural conditions and demands of each region, where training is specified by the CHW’s place of service. Some of these criteria, such as age, marital status, and level of education, may be also different, based on cultural and social conditions [13].

The CHWs in Iran are indigenous people, who are familiar with the local culture and have at least a high school diploma (with 12 years of general education). These people are selected after participating in a specialized test for becoming a CHW, including a written test and an interview. In Iran, the selection criteria for the CHWs are instructed by the MOH, and individuals who fulfill these requirements are granted permission to register in and enter the District CHW Training Center (DBTC). The selection criteria and instructions include some general and specific items. People with an associate’s degree or bachelor’s degree generally complete a six-month complimentary training course, while those with a high school diploma are required to participate in a two-year theoretical and practical training course to obtain a skill certificate [14–15].

Few studies have been conducted on the selection and recruitment of CHWs around the world, and there is no relevant study in Iran. Since different regions of Iran have different climatic, economic, cultural, and social conditions, it is essential to evaluate the selection process of CHWs and propose ideal criteria for selecting competent individuals, based on the conditions governing each region. The present study aimed to investigate the selection and employment processes of CHWs from the perspective of different strata of the community, based on the existing instructions to improve the effectiveness of healthcare delivery.

## Methods

### Study setting and participants

After obtaining ethical approval, this qualitative study was conducted in Golestan Province, Iran, based on all ethical and confidentiality principles. Golestan Province is situated in the northeast of Iran and shares borders with Turkmenistan. It has five active DBTCs, which are responsible for training students from all cities of the province. Three of these training centers have a reputation and a long history of training CHWs by skilled trainers with more than 15 years of experience. The participants were selected by purposeful sampling method among managers and supervisors of DBTCs, managers of healthcare centers, CHWs with at least two years of employment history in the main village where they work, and common people from the main village. Sampling continued until reaching data saturation (Table 1).

**Table 1:** Demographic characteristics of the participants

Participants	Number of participants	Age range (years)	gender	Employment history (years)	Educational level
Managers of the DBTC	M1	40–60	Female	30	Master’s degree
	M2		Female	28	Master’s degree
	M3		Female	27	Master’s degree
	M4		Female	24	Master’s degree
Supervisors of DBTC	m 1	40–50	Female	25	Bachelor’s degree
	m 2		Female	26	Bachelor’s degree
	m3		Female	25	Master’s degree
	m4		Female	29	Associate’s degree
	m5		Male	22	Bachelor’s degree
	6 m		Female	24	Bachelor’s degree
Managers of health centers	GK	30–50	Male	23	Master’s degree
	G1		Female	24	Associate’s degree
	G2		Female	20	Bachelor’s degree
Behvarzs(CHWs)	B1	46	Female	20	High school diploma
	B2	39	Female	18	High school diploma
	B3	32	Female	10	Bachelor’s degree
	B4	49	Female	25	High school diploma
	B5	32	Female	29	High school diploma
Common people of the villages(community)	P1	34	Female	-	Bachelor’s degree
	P2	31	Male	-	High school diploma
	P3	30	Female	-	Primary school
	P4	30	Male	-	High school diploma

### Study design

A qualitative thematic analysis was performed in this study. Thematic analysis, similar to other qualitative studies, is a method for describing and interpreting qualitative data in the process of identifying codes and building themes. It is a powerful and flexible method to understand experiences. Generally, thematic analysis is used in an extensive range of theoretical and epistemological frameworks for a broad range of study questions and sample sizes [16]. Several qualitative thematic analysis models were identified during this study. Brown’s thematic analysis model is one of the methods widely used today. In this model, the researcher does not follow a linear path of analysis; instead, an iterative approach using the interview transcripts, codes, and themes continues until obtaining the desired outcomes [16–17].

### Data collection

Data were collected using semi-structured interviews with all stakeholders engaged in this study during 2021. Before conducting the interviews, a consent form was completed and signed by the respondents. They were also free to withdraw from the study at any time and were assured that the content of the interviews would be kept confidential in a secure place. The average time of each interview was 45 min. Interviews were conducted using an interview guide, consisting of several key open-ended questions:

- How would you like the CHWs who work at your place of service to act? (responded by common people)
- Do CHWs working at your place of service have enough skills to perform their duties? (responded by all participants)
- Are the current methods of selecting and hiring CHWs at your place of service effective in recruiting sufficiently skilled individuals? (responded by all participants)
- Do you think that the criteria for choosing CHWs in your community should change? Please mention your intended items (responded by all participants).
- What items in the guidelines cause qualified people to be dismissed from work? (responded by managers and supervisors)

The interviews were recorded and then transcribed verbatim by the researchers.

### Data analysis

The collected data were analyzed by the qualitative thematic analysis method using an inductive-deductive approach, based on the six-step model by Brown and Clark (2006). The first step (i.e., being deeply acquainted with the data) was performed by reading the transcripts several times. At this stage, the transcripts of the interviews were read several times to become adequately familiar with them and to extract primary concepts and codes. In the next step, the transcripts were organized into meaningful concepts to extract primary open codes from the data, representing all the introduced concepts. Subsequently, primary themes were extracted after establishing a deep relationship between the transcripts and codes, and the researchers tried to move forward and reflect on the concepts and codes to search for themes at both macro (e.g., wide and general concepts) and micro (e.g., all concepts of codes extracted) levels. Finally, using an inductive process, relationships between themes and codes were identified and established. Next, the themes and sub-themes were named and interpreted. The relationships between codes and themes in each section are shown in tables and graphs. Moreover, relationships between family codes, sub-themes, themes, and primary open codes were represented using ATLAS.ti version 8.

## Results

The demographic characteristics of the participants are shown in Table 1. Data saturation was achieved after interviewing seven managers, six supervisors, five CHWs, and four common people.

**Fig. 1:**
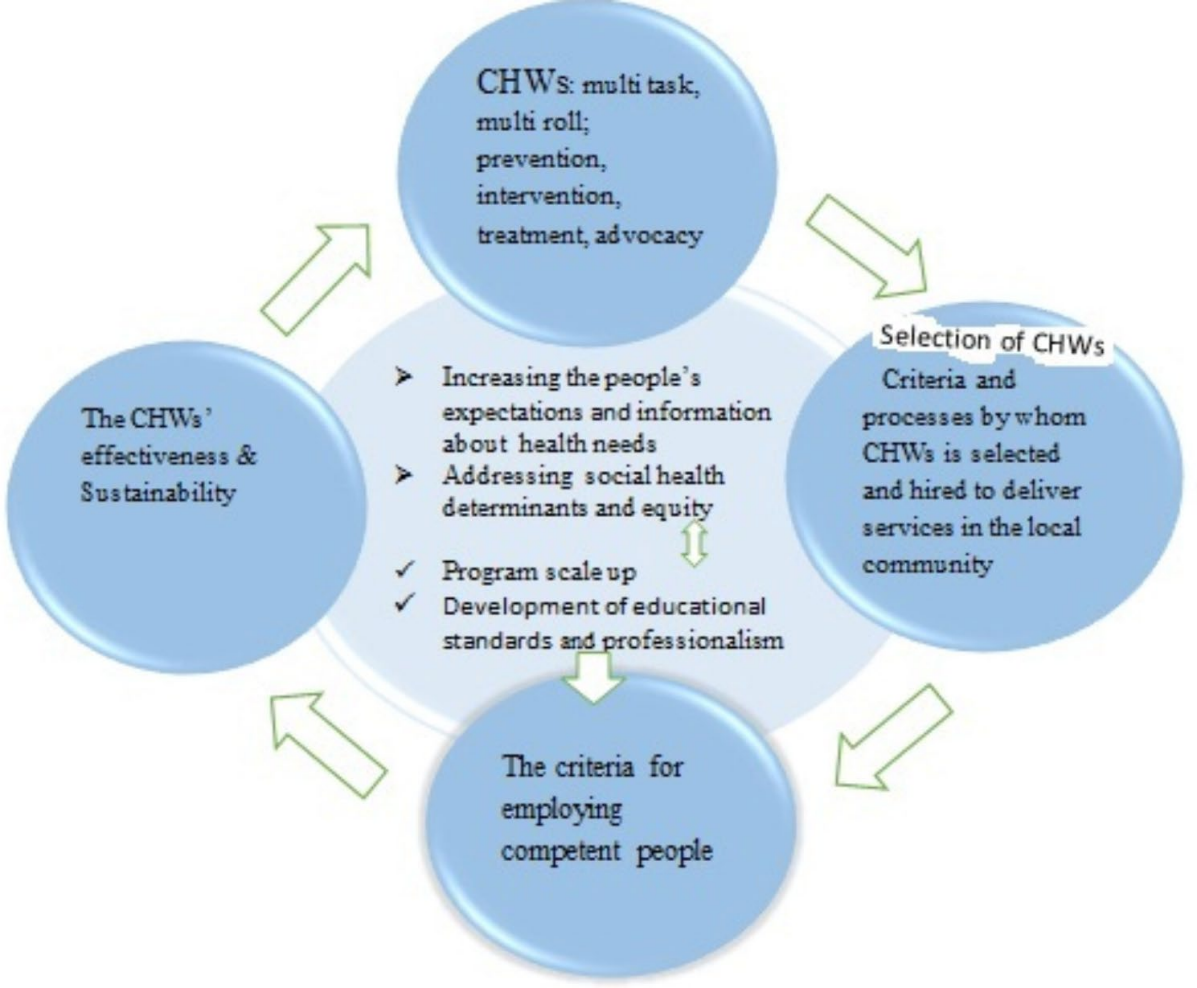
A summary of research findings at the macro level

An initial overview of the primary open codes and the participants’ statements raised a general question at the macro level: “What are the criteria for the selection and employment of CHWs?” According to the findings, criteria for selecting and hiring these individuals should be defined in a way that they can fulfill their tasks and roles in areas under coverage. Attention to these criteria helps the CHWs to fulfill their tasks and enables them to effectively perform their roles in the community. A deeper look into the transcripts of the interviews and codes, extracted from the common people’s statements, revealed that they preferred to have intelligent CHWs with higher education levels and credible certificates, who could perform difficult tasks. Additionally, they found that CHWs should have the basic knowledge for necessary training. Figure 1 summarizes our findings at the macro level.

**Fig. 2:**
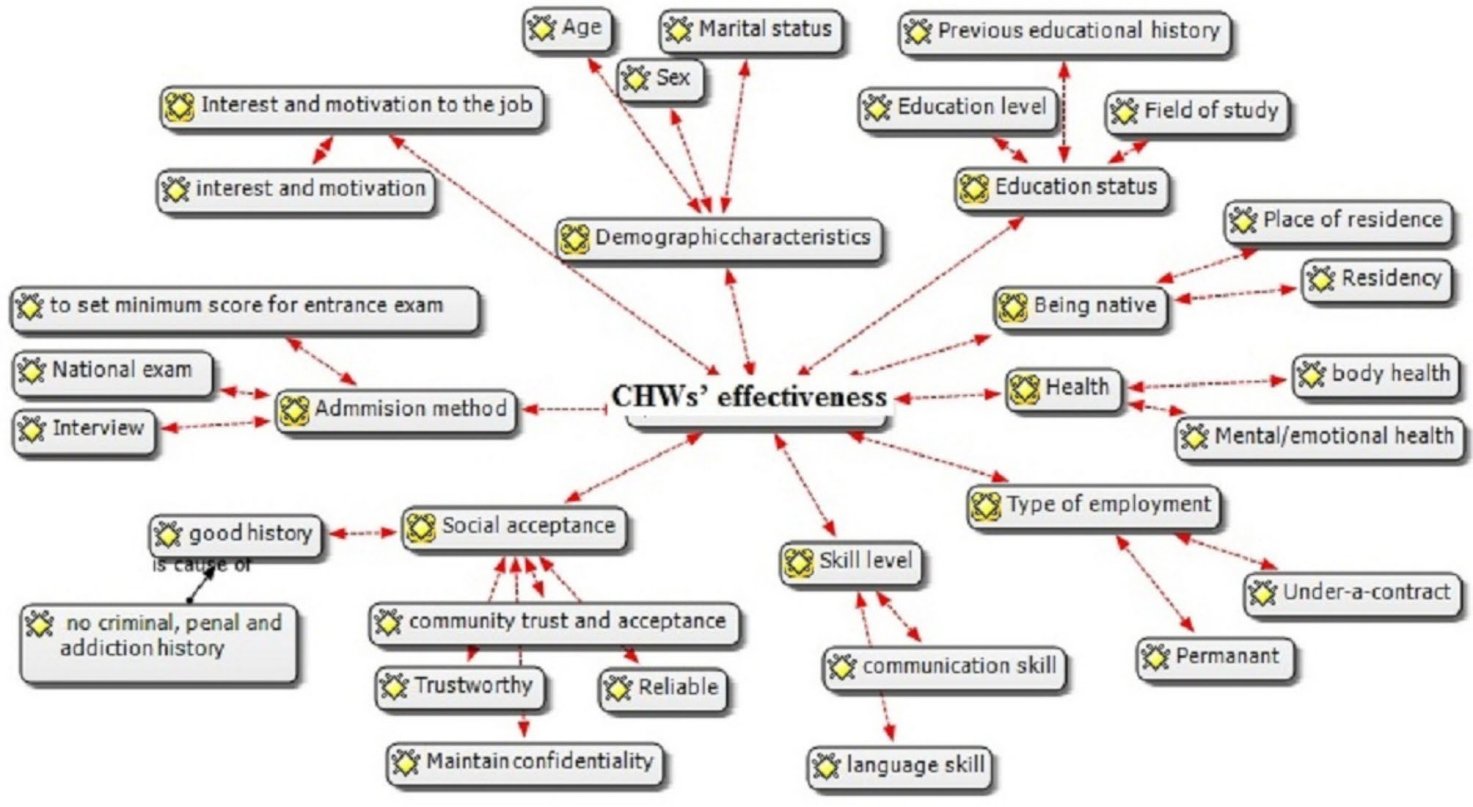
Summary of the CHW effectiveness and related codes

The codes and interview transcripts were reviewed several times, leading to the emergence of 340 primary open codes, which were reduced to 115 after re-evaluation and merging of overlapping codes. Two main sub-themes, that is, “CHW effectiveness” and “CHW sustainability” were derived from the extracted categories and codes. Finally, the main themes were identified as “criteria for employing competent people”, “barriers to employing competent people”, and “identifying the barriers to employing competent people” in the instructions. Figure 2 presents the sub-theme of CHW effectiveness and the extracted codes for selecting qualified people and providing highly effective services from the participants’ perspective.

### Criteria for employing competent people

These criteria could lead to the delivery of effective health services, according to the participants’ viewpoints. One of these criteria fell into the category of “specific admission codes”, encompassing the subcategories of “educational status” (i.e., type and rank of prior education and history of educational performance), “admission method” (i.e., admission through the national entrance exam or a regional test, acquiring the required credit score, and participating in an interview), “type of employment” (contractual or permanent), “amendments of employment acts”, and “interview process”. An excerpt of the statements of various stakeholders participating in this study is presented below.

### Entrance exam and interview

In this regard, CHW No. 1 stated:

*I suggest that instead of hiring experts through an employment exam, it should be based on the national exam, and training should be entrusted to health worker training centers so that educated people are employed more frequently and are trained more efficiently.*

Also, one of the DBTC managers (DBTC No. 1 manager) stated:

*I suggest hiring people in a decentralized manner, based on a national entrance exam by giving extra credits to those who live in cities rather than villagers.*

“*I disagree with hiring people without a test; so, if a native villager cannot obtain the minimum score, those who can acquire the required score should be selected in the vicinity of villages.”* (CHW No. 3).

“*Since there was no required minimum score, those obtaining the highest score would be accepted. The highest score in one village might be 80 out of 100, while in another village, it might be 5 out of 100. Naturally, we have to deal with highly illiterate people and try very hard to help them graduate.”* (Supervisor of DBTC No. 5).

Another frequently stated code was the necessity of amending the interview process and using experienced interviewers. Most of the participants believed that the interview process and its scoring methodology should be corrected, considering the stronger role of interviews in hiring and admission. In this regard, one of the managers of health centers (No. 2) stated:

*The interview items should be clearly specified*...

“*Experts involved in the interview lack enough experience and competency for diagnosis of mental disorders.*” (Supervisor of DBTC No. 3).

### Primary educational level

Based on the findings, hiring people with high academic levels could be beneficial, as they performed better when communicating with the formal sector of the society. In this regard, one of the managers of health centers (No. 1) stated:

...*I think that the higher one’s academic level is, the better their interaction will be with middle-rank sectors, that is, comprehensive community health centers. The quality of our work will also increase*. *New information is emerging every day. Old-fashioned health workers cannot learn integrated contents. They simply fail to learn such information.*

The common people of village No. 1 also said:

...*It is important that they care for the people they treat and have a high level of literacy.*

Also, one of the managers of health centers (No. 1) stated:

*People prefer to see educated people working at health centers and ask for more specialized people to be available at these centers. For example, they prefer to have a midwife in the health center so that they don’t have to travel to the city, even if the distance is not so long.*

Another extracted code was the previous educational degree, which was assumed to be the basis for increasing CHW training preparedness from the viewpoint of managers and supervisors. The majority of managers and supervisors believed that CHWs should be selected among people with high school diplomas in experimental, or at least mathematical, sciences. In this regard, the supervisor of DBTC No. 4 stated:

*We’ve had people who were accepted based on this rule and had a vocational diploma with an average of 11 or less (out of 20). We have problems teaching them the simplest biological concepts about the body or even other topics.*

The person’s school performance was one of the most frequent topics described by the majority of managers and supervisors. The supervisor of DBTC No. 3 stated:

*Successful community health workers were usually good students from elementary school to high school. I mean people who were successful in their education before being registered for the healthcare course became also successful in their job. Good students usually turn into good health workers.*

### Personal qualities

The codes related to the category of “physical and mental health” were among the most repeated items in the participants’ statements. They emphasized that the CHWs should have high physical and psychological health, since providing community health services is a difficult task; for instance, people excluded from the military service due to medical reasons should not be recruited as a CHW. The participants also believed that psychological and mental health should be evaluated using standard methods. The manager of DBTC No. 2 stated:

*Because health workers face many difficulties in times of crisis, they need to be healthy. So, a person excluded from the military service for a medical reason, such as vision or hearing problems, is not eligible.*

Moreover, the manager of DBTC No. 1 said:

*Rather than asking a few simple questions with simple answers during the interview to confirm the applicants’ mental health, it is better to complete a standard questionnaire to better evaluate their psychological health; in this way, health providers are not selected based on only higher scores.*

Additionally, the manager of DBTC No. 3 stated:

“*I see community health workers who would not be in this position if they had been chosen more strictly at the time of hiring…I have seen health workers, for example, a man in his thirties, crying because of his high work pressure. This means that he is not suitable for this job; maybe, if he worked in another department dealing with administrative affairs, he would have been more successful.”*

The category of social acceptance included repetitive codes, such as safe background records (e.g., no criminal and penal records or addiction) and previous employment history to establish social trust and approval. Moreover, all participants highlighted principles, such as respecting confidentiality, support, and honesty as fundamental characteristics to achieve social acceptance. The manager of DBTC No. 2 said:

...*It is very important for a person who wants to work in a health center to not have a criminal record so that people can trust them better; they should also have social communication skills so that others can accept them.*

Adherence to the principles of confidentiality, support, and fair delivery of services were among parameters described by the public. Some of the participants stated that they would not share or discuss their family problems or psychological issues with the CHWs. Common people of village No. 3 said:

*I do not usually talk to or confide in health workers about my family or mental issues. I only go to them to control my own health or the health of my child.*

Moreover, CHW No. 5 said:

*Sometimes, people share their private issues more easily with a non-indigenous person who does not live in their village and has no family relations with the villagers, because they would not have to worry about the spread of their private issues. I am a non-indigenous health worker; so, some people trust me more.*

The participants believed that the candidates’ interest and positive attitude toward their job should be evaluated. The supervisor of DBTC No. 2 stated:

*Usually, people who ask about the duties and responsibilities of community health workers before registration and make an informed choice adapt better to their job and become more interested in it.*

The manager of DBTC No. 1 said:

*At the time of admission, practical tests for special skills, such as communication or language skills, should be included.*

### Type of employment

The type of employment (permanent or contractual) was stated as a factor contributing to the self-learning and self-improvement of CHWs. The manager of DBTC No. 4 stated:

*It is good to hire people on a contract. I believe that this is beneficial as people understand that they are being regularly evaluated, and therefore, they keep themselves up-to-date.*

### Age, sex, and marital status

Some demographic characteristics, such as age, sex, being indigenous, and being married, were among repeatedly described codes and important acceptance criteria. Besides, age was an important criterion for hiring the CHWs. Older people have poor learning abilities and are incapable of hard work or traveling frequently. They are also more likely to retire early. In this regard, the manager of DBTC No. 2 stated:

*The age requirement should be one of the criteria for acceptance as health worker, because these people are supposed to work for 30 years, and at older age, the learning ability declines; even the age of 28 years may be a very high threshold for age. The elderly are not as efficient as young people and are subject to early burnout.*

Female gender and being married were among criteria for the CHW employment, boosting the effectiveness of service delivery and service provision. In this regard, CHW No. 4 stated:

*Men are usually less interested in and serious about this job, which is mostly due to their low income, forcing them to seek other jobs. In my opinion, female community health workers have higher work conscientiousness and seriousness.*

Additionally, CHW No. 2 stated:

*People who are educated, married, and interested in their job are more successful.*

### Local hiring and residence

Local hiring and residence in villages were among the most repeated codes. Factors discouraging people from staying in their village and motivating them to leave their workplace included children’s education, marriage, and seeking a higher position, causing them to migrate to the city; therefore, CHWs need to travel back and forth to the village to provide services. In this regard, CHW No. 1 said:

*The likelihood of community health workers to permanently stay in their villages is substantially low, because their children’s education is an important issue for them.*

Most of the participants agreed with the necessity of expanding the scope of local hiring, while few of them agreed that all volunteers should come from the target village. The manager of DBTC No. 4 stated:

*If there are people with relevant academic degrees in the village, native people should be prioritized, but if there are no natives with relevant education in the village, it is necessary to expand local hiring to cover the surrounding villages or cities, as well.*

The manager of DBTC No. 4 stated:

*Because we had three applicants with diplomas, we could not register more talented young people from a satellite village. I even know that the hired workers had many problems during school years.*

One of the challenging issues was discrimination in informing and providing services in health centers to the families of CHWs. Common people of village No. 2 stated:

*In my opinion, family favoritism is one of the reasons why we do not know what services have been delivered by the health center. They treat their own families better and inform their relatives about everything sooner.*

Factors, such as marriage, residence, interest, social acceptance, and good performance, were among the most effective contributors to sustainability and retention.

### Barriers to employing competent people, comparison with instructions, and proposal of solutions

Table 2 shows conflicts between the MOH instructions and the viewpoints of managers and supervisors. In this section, the clauses of instructions were reviewed and compared with themes, sub-themes, and codes extracted in this study. The conflicts and solutions proposed by the participants (managers and supervisors of DBTCs) were identified, as shown in Table 2, by presenting examples of the concepts.

**Table 2:** Barriers to the employment of qualified people (contradictions between the participants’ viewpoints and instructions)

Conflicting codes with the MOH instructions	Basic sections (supervisors and managers’ viewpoints)	Suggestions for improvement
**Instruction problems**	“*The Ministry of Health manual has many pitfalls that need to be eliminated. Some of the specific or general terms restrict the recruitment of qualified people*.”	The instructions should be further revised.
**Non-specification of the field of study (high school diploma) in the MOH instructions**	*“…Employment of health workers from any field of study leads to the recruitment of low-literate people by training centers.”*	Most managers and supervisors believed that CHWs should be selected among people holding diplomas in experimental, or at least mathematical sciences.
**Absence of a passing score on the admission test for native candidates (from the main village)**	*“…The admission process does not have a minimum score, and volunteers with low general literacy levels are accepted. The presence of people with different literacy levels in the same class causes teaching and learning problems…”*	If a native villager does not obtain the minimum score, people who have acquired the required score should be selected from nearby villages and other places. (Revision of Note 4 in the instructions)
**Inattention to previous academic records**	*“…There should be a requirement for having a high school diploma, because people with a low average score experience problems in learning high school courses; things will be even more difficult in community health training courses.”*	Performance records in high school graduates should be assessed.
**Type of** **employment**	*“…It is preferred to hire people on contract. I believe that this is beneficial, as people know that they are being regularly evaluated; so, they keep themselves up-to-date.”*	Contract employment is preferred over permanent employment.
**Challenges of selecting native people and local hiring**	*“I believe that health workers should be hired in a decentralized manner, based on a national entrance exam by giving extra credits to the natives of cities rather than villages.”*	The concept of local hiring (e.g., extending the scope of local hiring from villages to cities and registering candidates at the province level rather than the village level) should be assessed.
	*“…Natives have incomplete information in large villages, and the village proximity to the city reduces cultural differences among people…”*	
	*“…We could not register more talented young people from nearby villages.”* (Note 4 of the MOH instructions)	
	*“…The likelihood of a community health worker to stay in the village forever is substantially low…”*	
	*“In our region, there is no difference in the cultural status of villagers and urban residents, because most cities are newly developed due to the migration of rural people.”*	
	*“Sometimes, people share their private issues more easily with a non-indigenous person who does not live in the village and has no family relations with the people of that village, because they do not have to worry about the confidentiality of their information.”*	
**Effects of marital status on the CHW’s stay in the village**	*“The acceptance likelihood of married people is higher, because their residence status is known. If they want to hire native people, they should be married and live in the village so that they don’t immediately ask for a transfer.”*	Married people should be prioritized as CHWs.
**Unclear interview process**	*“…The interview items should be clearly specified…”* *“Experts present in the interview lack enough experience and competency for diagnosis of mental disorders.”*	The interview process, rules, and regulations should be amended, and experienced interviewers should be recruited.
**Absence of a standard method for the assessment of candidates’ mental and psychological health**	*“…They are not properly examined in terms of personality and psychological issues. Any person with any psychological and physical characteristics can be accepted…”*	The mental and psychological status of candidates should be examined using standard tests.
**Non-transparency of items related to criminal convictions and offences**	*“…Criminal convictions and penalties should also have specific items in the exam. Certainly, a person who has a legal violation problem cannot be a suitable health worker, but it is different when a person has a legal problem, due to, for example, an unintentional car accident.”*	Certain items should be set for identifying the candidates’ criminal convictions.
**Failure to assess the candidate’s interest in job**	*“People who are interested in their job and participate in the entrance exam work just as hard until retirement, whereas people who enter with quotas or because of family coercion or unemployment are not successful.”*	Interest in providing community health services should be assessed.
**Failure to assess the candidate’s communication and** language skill**s**	*“…Be friendly, give good guidance; expression is very important.”*	Communication and language skills should be assessed.
**Recruitment of incompetent CHWs**	*“Quotas prevent the recruitment of qualified people, because they are accepted without any evaluation.”*	People with quotas should be assessed the same way as other volunteers.

MOH: Ministry of Health; CHW: Community health worker

## Discussion

In many countries around the world, community health workers play a unique role in establishing a link between the healthcare system and society. Today, development and transformation of social health needs have resulted in the development of health programs and management of unpredictable cases. Therefore, selection and employment of suitable people who can fulfill the assigned tasks and roles and also meet the needs of the society are among the main priorities of the healthcare system.

The present study explored the perceptions and experiences of health system stakeholders at different levels, based on the criteria used for selecting and employing CHWs to help identify qualified people and provide effective community-based services. The present findings are promising due to the localization of employment criteria in order to select highly qualified people. Moreover, instructional barriers to selecting competent people were highlighted according to the perspectives and experiences of DBTC managers and supervisors (Table 2). The exploratory themes extracted in this study and their relationships are presented as concepts and sub-themes.

### General and personal criteria

Some of the general requirements based on the MOH instructions are the lack of addiction to cigarettes, narcotics, and psychotropic substances, lack of a history of criminal offenses or convictions, having physical and psychological health, and not being banned from employment in governmental institutions according to legal organizations. The findings of the present study showed that all the participants had a positive attitude toward the general requirements in the MOH instructions. However, the some sub-themes identified in this study did not fulfill some of these general requirements; these sub-themes could not be evaluated when completing the registration form.

The participants believed that providing community health services was one of the difficult tasks of the CHWs, who were required to have physical and psychological agility. Some individuals may choose this occupation just to have a source of income and may be less motivated, which contributes to their poor performance. All the participants believed that the CHWs should be interested in their job, besides being compassionate, inspired, reliable, responsive, and sociable. An ample amount of evidence suggests that these features should be considered when employing people as social service providers [18–19]. Studies have also shown that confidentiality and poor trust are among key barriers to hiring CHWs who can provide maternal and pediatric health services [20].

In a previous review, the most important criteria for the employment of CHWs were their personal characteristics, such as interest in the field, willingness to learn, and compassion. It has been also reported that non-financial incentives, such as trust, respect, familiarity with the community, and self-esteem, are among the potential triggers of favorable performance. In this regard, Mishra et al., in a study in India, explained that CHWs should be more interested in their job than money [21–23].

### Primary educational level

The primary education level is one of the specific requirements for being accepted as a CHW; therefore, all candidates need to have at least 12 years of academic study [15]. According to the sub-themes identified in this study, the participants believed that the quantity and quality of education were associated with competency-based selection. Common people stated that the CHWs should have a high level of education to be trusted. Interestingly, common people also cared about the university where the CHWs had studied.

According to the supervisors and managers of the CHW training schools, a person’s basic education, including the field of study and academic records, was an important criterion. They believed that people graduating with a diploma in experimental sciences were better prepared to receive the necessary health education and were usually more successful than their graduated counterparts. Another important background factor was the person’s records in their last educational course. People with poor academic records usually face more problems in the CHW training courses. The importance of the basic educational level of CHWs, as highlighted in this study, has been also reported in previous research. Evidence shows that the CHWs’ inadequate education or certifications can negatively influence the community’s belief about their effective response and reaction to their needs. Besides, CHWs with higher educational levels may become convinced to quit their job after being employed [22, 24–25].

In Uganda, the community had lower acceptance for people with low education levels [26]. In Brazil, having a formal educational degree boosted the acceptance of CHWs in the society, as well as people’s trust in them. Moreover, the professional activity of CHWs in cooperation with other health workers increased their social credibility [27]. A study in Bangladesh showed that less literate people did not receive the required training for their occupation, contributing to their poor functionality [25–28]. However, conflicts and instability in the work environment of CHWs generally impede recruiting people with a specific level of education. This challenge is heightened in critical situations, such as the outbreak of diseases, or when there is a shortage of human resources [24, 29–30].

According to studies in some countries, people do not believe that the CHWs should have a basic level of literacy, as they can receive the necessary training after employment. Evaluation of healthcare delivery by illiterate and less literate people in countries, such as Nepal, shows that these individuals receive effective and need-oriented training by their supervisors after employment [13, 31]. A study concluded that completion of primary school education should be considered as the minimum educational requirement for the CHWs to meet the care needs of underprivileged and remote communities [32–33].

### Type of employment

In Iran, the CHWs are employed by the government and are paid monthly, which is a relatively strong motive for entering this occupation and performing well [15]. The findings of this study showed that the type of employment (contractual or permanent) was an influential factor in the CHWs’ motivation for self-improvement and self-learning to remain in the system. The participants of this study believed that people who were hired officially and permanently had less motivation for self-improvement and delivery of quality services. However, the CHWs on contract tried to exhibit better performance to maintain their status and acquire the approval of the system. Ample evidence suggests that financial incentives are among factors improving the performance of CHWs. Today, health systems are moving in this direction to increase the CHWs’ motivation and durability in the system by offering them financial advantages [34].

### Gender and marital status

According to national instructions, one female CHW and if required, one male CHW should be employed for every 1000 villagers. Based on the findings of this study, the participants assumed that women and married CHWs would provide more efficient services. In various countries, such as India, Brazil, Pakistan, and Nepal, which have a reputation for community-based plans, two factors, that is, female gender and being married, were the main criteria for hiring the CHWs. In these countries, community-based services focused on the family, children, and women and were tailored to the cultural framework; also, stranger men were not allowed to enter people’s homes [13].

Generally, recruitment of female CHWs is challenging in countries, such as Afghanistan, where women are not allowed to travel unattended; therefore, one woman and one man are always selected as CHWs to provide healthcare services. Similarly, in Kenya, both males and females were recruited for this purpose [13, 15, 35]. Since some services are more easily delivered by females in some countries, women are mobilized and encouraged to choose this occupation. In some underprivileged communities, the CHWs are selected among women for empowerment [30, 36].

### Age

The MOH instructions have determined a specific age range for employing the CHWs; the maximum age should not exceed 30 years. According to the codes extracted in the present study, age was recognized as an influential criterion in the effectiveness of services provided, learning ability, and capability to fulfill responsibilities and duties defined for the CHWs. Findings show that age is an important factor in hiring the CHWs in many countries, where a certain age range has been specified [13, 15].

### Local hiring and residence

In many countries, selection of CHWs from the community where they are expected to serve is one of the basic principles for providing community-based services; this principle has been strongly recommended by the WHO [13, 15]. In the rural regions of Iran, selection of CHWs is strictly based on the WHO recommendations (i.e., selection of CHWs from the community where the service is to be delivered); this is one of the specific criteria that candidates need to fulfill before registration. According to the instructions, if there are enough candidates (usually three people with High school diplomas) from the main village, they will be selected and hired after passing the exam and interview. And if there is not enough candidate from the main village, candidates from the neighbouring villages can register to take the exam and get hired [15].

One of the challenges of DBTC managers and supervisors was that candidates coming from the main village did not obtain the required credit score in the written entrance exam (i.e., selection of incompetent people). Moreover, the present findings showed that inflexible adherence to the core instructions of MOH, besides the restricted selection of candidates from the main village, hindered the employment of qualified people from nearby villages or other areas. Overall, it is important to explore the perceptions of stakeholders, including the managers and supervisors of DBTCs (as service providers) and the society (as a service demander). The managers stated that employing incompetent people caused many workers to be either fired from their job due to poor performance or resign themselves; this turnover imposed high costs on the system and led to the waste of resources. Supervisors also complained about the difficulty of teaching people with low capabilities.

Evaluation of common people’s perception showed that they did not care if the selected CHW was a native of the village or not. Instead, they preferred highly literate and skilled people for employment to support them and respond to their needs; substantial evidence from different countries supports this finding. According to previous research, the CHWs, by participating in the community they serve, can mobilize the community to improve a wide range of health practices. However, this goal is only achievable when the community has a positive attitude toward the CHWs and accepts them. Studies show that many parameters can affect the social acceptance of CHWs. The priority of these factors may vary in different societies, depending on the cultural, economic, and climatic conditions. The critical importance of this parameter lies in the acceptance of CHWs by the community where they serve, besides increasing the motivation, responsiveness, and accountability of the CHWs [24, 37]. A report from Ghana and Rwanda indicated that being chosen by the community boosted the CHWs’ sense of responsibility, motivation, and pride when fulfilling their roles [38–39].

However, outcomes vary from one country to another, as selection does not always proceed according to the instructions. The results of an ethnographic study by Rafiq et al. in Tanzania, investigating the relationship between professional outcomes and the CHWs’ personal and social identity, showed that distinguishing personal identity from professional identity was difficult in CHWs working in rural regions. This study also demonstrated that the CHWs’ personal identity sometimes prevented them from talking about issues related to family planning and sexual health [40]. Moreover, the findings of a study from Kenya revealed that CHWs, selected by the community, as well as those whose selection was not related to the community, showed similar adherence to the instructions [41]. Besides, reports from India and Ethiopia indicated that the CHWs were selected without seeking the community’s opinion; however, in Uganda, some community members preferred non-natives as CHWs [42–43].

The results of the present study revealed that people sometimes avoided sharing their physical and psychological problems with native CHWs due to concerns over confidentiality issues, fear of information disclosure, and social stigma. Several studies from different countries reported problems when hiring natives in places where there was stigma over a certain disease, for example, AIDS in African countries. Therefore, it is preferable to employ non-native CHWs for these people and sometimes for male immigrants [33, 44–45].

Discrimination in offering services and paying special attention to relatives by native CHWs were among factors that challenged the community’s trust in them; nevertheless, findings are controversial. Some studies carried out in different countries have reported the positive effects of kinship ties on the professional roles of CHWs working in rural communities. Other studies in Nigeria and South Africa have also confirmed the central role of kinship and self-identity in the positive and trusting relationship nurtured between the CHWs and the community where they work [33, 46]. The results of an ethnographic study in Tanzania also revealed that the use of kinship terms, such as father and mother for male and female CHWs, could facilitate the interaction between personal and shared roles and professional duties to build trust and a sense of ownership in health-related programs [40].

On the other hand, the community’s participation in the selection of CHWs can also cause several problems. When the selection process is not transparent, there may be misinterpretations in supporting certain groups, which can hurt the community emotionally and make them lose the spirit of cooperation. When the selection of CHWs is managed by traditional kinship structures, despite the increased social participation and effectiveness of interventions in the kinship group, it can lead to the exclusion of other community groups and discrimination against them. Conflicting findings have been reported in Uganda and India [47, 48].

Based on previous findings, local hiring and increased access to health services may not always yield desirable outcomes. Many factors related to both suppliers and demanders should be addressed to achieve equity. According to previous studies, deprived and underprivileged groups are usually less capable of adhering to the CHWs’ recommendations due to economic and non-economic reasons [37, 45, 49].

Some findings suggest that selection of CHWs from communities living in suburb and remote areas can improve access to health services. Also, according to previous reviews, selection and employment of low-educated CHWs from communities with low literacy levels or poor people in poor communities can improve access to health services and lead to the fair distribution of these services. Moreover, home visits can be helpful for people who are prevented from visiting health centers due to cultural obstacles. The engagement of traditional healers can help provide services to groups with certain cultural traditions [45, 49].

Based on the findings of the current study at the macro level and exploration of concepts and codes extracted at the micro level, it is obvious that the society’s expectations of service providers are increasing, the importance of attention to the health of villagers, being supportive, providing high-quality services, and CHWs’ ability to provide new and diverse services. The participants also believed that CHWs with higher levels of literacy and capability could perform better in establishing vertical equity and mobilizing at formal levels [37]. The present findings about CHWs indicate the expansion of professionalization, increased service quality in scale and variety, transforming roles, and coping with unpredictable situations [24]. In recent years, professionalization has expanded due to increased interactions between communities and health systems and increasing demands for diverse, high-quality, and up-to-date services, provided by more professional individuals [50, 52–55].

For health programs to be effective, selection and employment of CHWs should be tailored to the society’s needs and underlying conditions. Even in a single country, the criteria for selecting CHWs may vary from one place to another. Therefore, CHW recruitment programs are recommended in various regions for comparison to optimize relevant policies; otherwise, challenges are unavoidable. Therefore, it is important for managers to show flexibility to upgrade and expand the CHW recruitment strategies [13, 33].

### Limitations

Iran is a country with great diversity in terms of climatic, geographic, cultural, social, and economic conditions in different areas. In some regions of the country, villages are far from cities and other villages, while in some regions, cities and villages are interconnected; therefore, people living in different regions may have variable access to facilities. The present study was conducted in Golestan Province in north of Iran; therefore, generalization of the findings to other parts of the country with different backgrounds may be challenging. It is necessary to modify the CHW selection and recruitment criteria in a way that guarantees the employment of most suitable individuals who can fulfill the assigned roles, address the community’s needs and demands at the place of service, and minimize depreciation in the system.

One of the limitations of this study was the small number of samples. Although, based on the current guidelines sample size for qualitative studies is varied. Data saturation is a significant measure to determine a sufficient sample size in qualitative studies. Given that each study has a unique characteristic and the saturation point can vary, it is also possible that no data is truly saturated. In any case, the study should be carried out with more samples to discover the dimensions of the subject [56].

## Conclusion

The CHWs act as mediators between the society and the formal sector of the healthcare system, which provides effective services to upgrade social health. The findings of this study showed that the more capable and literate these people are, the better they can fulfill their roles. Therefore, selection and recruitment policies should be based on competency; it is also certain that some of the current CHW recruitment criteria in Iran impede competent selection.

According to the aforementioned and specific conditions of each region, competent selection should not be overlooked in the favor of local hiring. Nevertheless, it may not be suitable to establish specific identical selection criteria for all regions of the country, and it is preferred to localize these criteria based on the condition of each region to achieve justice in the healthcare domain. Besides, when selecting the CHWs, sufficient attention should be paid to factors, such as interest, social trust, acceptance, ability to acquire necessary training, ability to fulfill duties, and physical and psychological preparedness.

## Data Availability

The datasets generated and/or analyzed during the current study are not publicly available due [Given that the data was collected through interviews. Opinions and views of people are confidential.] But are available from the corresponding author on reasonable request.

## Abbreviations

DBTC: District Behvarz Training Center
MOH: The Ministry of Health
MOHME: The Ministry of Health and Medical Education
WHO: The World Health Organization
CHWs: Community Health Workers
